# Eliminating dark-rim artifacts in first-pass myocardial perfusion imaging

**DOI:** 10.1186/1532-429X-15-S1-O3

**Published:** 2013-01-30

**Authors:** Behzad Sharif, Rohan Dharmakumar, Troy LaBounty, Chrisandra Shufelt, Louise E Thomson, Noel Bairey Merz, Daniel S Berman, Debiao Li

**Affiliations:** 1 Biomedical Imaging Research Institute, Cedars-Sinai Medical Center, Los Angeles, CA, USA; 2 Heart Institute, Cedars-Sinai Medical Center, Los Angeles, CA, USA

## Background

**Figure 1:**
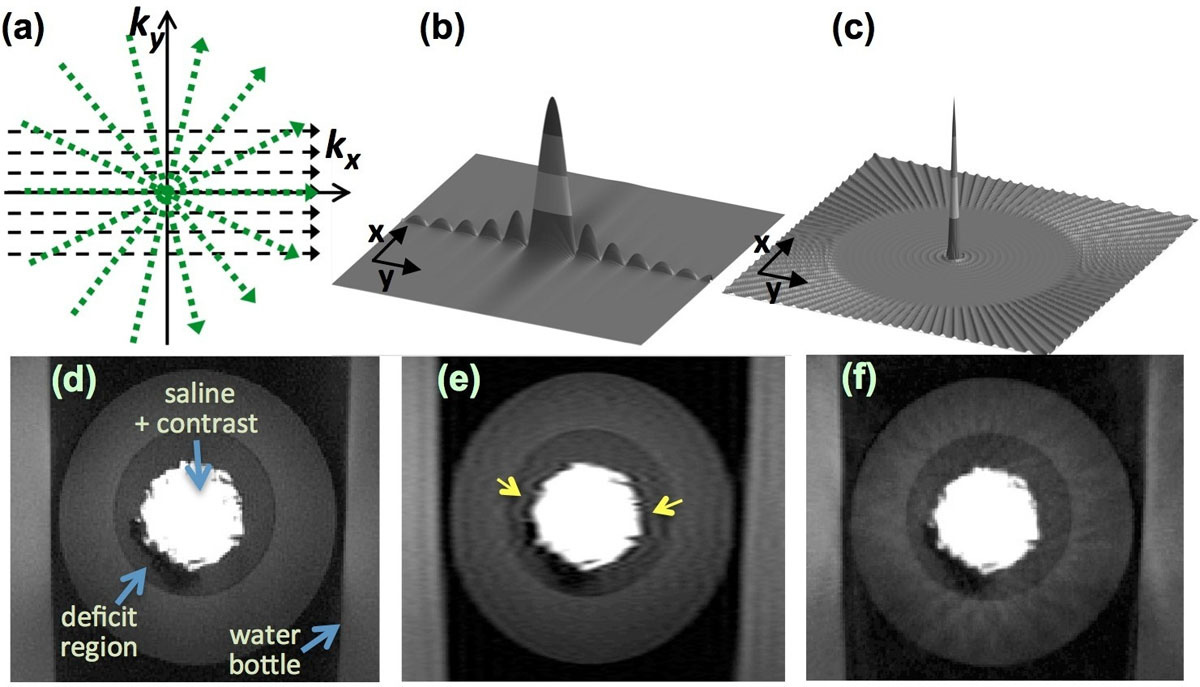
(a) Cartesian and radial k-space sampling patterns with the same number of readouts and readout resolution; (b) corresponding point-spread function (absolute value of the PSF) in image domain for Cartesian acquisition; (c) PSF for radial acquisition. Insufficient k-space coverage along Ky (phase-encode direction) results in significant ringing along y, as shown in (b). Panels (d)-(f) show reconstructions of an MR gelatin-Gadolinium phantom with realistic signal intensity ratios, demonstrating robustness of projection imaging to Gibbs ringing: (d) fully sampled (ground truth) image with 1x1 mm resolution (384x384 matrix); (e) Cartesian imaging with 108 phase-encodes (arrows point to DRA); (f) radial imaging with 108 projections (no DRAs, mild streaking).

We demonstrate that projection imaging significantly reduces the prevalence and spatial extent of subendocardial dark-rim artifacts (DRAs) in first-pass perfusion (FPP) myocardial MR, compared to conventional Cartesian techniques. A major cause of DRAs, which remain a major concern in FPP imaging, is known to be the so-called Gibbs ringing (truncation) phenomenon [1-3]. Radial k-space sampling exhibits minimal Gibbs effects with typical FPP parameters, thereby eliminating a major contributing factor to DRAs [4]. The underlying theory is demonstrated in Fig. 1, which describes Cartesian and radial k-space sampling (with the same number of readouts) and the corresponding point spread functions (PSFs). Insufficient coverage along phase-encode direction with Cartesian sampling results in significant ringing in image domain (Fig. 1b). In contrast, angular undersampling results in streaks outside of a "local" region for radial images (Fig. 1c). Panels 1d-f show phantom studies (Gelatin-based with realistic contrast ratios, resembling the LV with a deficit region) verifying the described PSF effects.

## Methods

Healthy human volunteers (N=12) were imaged on a 3T scanner (Siemens Verio). Two FPP scans (SR-prepared FLASH) were performed at rest (>10 minutes gap) using a single-shot radial pulse sequence followed by a single-shot Cartesian sequence (common parameters: FOV read =270-350 mm; BW ≈800 Hz/pixel; flip angle = 12°; TR =2.4-2.6 ms; TI =100 ms). Both scans were accelerated using rate 2 parallel imaging (TGRAPPA for Cartesian and SENSE for radial) and the number of readouts per frame was matched within 10% (range: 48-56). Scans were visually read for artifact by 2 expert readers blinded to the study protocol using a consensus 0-4 scoring scheme (0:no DRA; 4:severe DRA).

## Results

**Figure 2:**
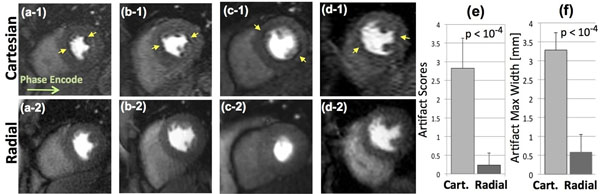
Representative first-pass CMR perfusion images from 4 of the 12 studied healthy humans: top Panels (a-1) to (d-1) show Cartesian images (arrows point to DRAs); Bottom Panels (a-2) to (d-2) show the corresponding radial images. All images correspond to similar early myocardial enhancement phase (7 heart beats after initial LV contrast uptake). Panel (e) shows summary of artifact scores assigned by two expert readers (consensus 0-4 scale scoring, 0: no DRA, 4: severe DRA). Panel (f) shows the maximum measured width of the artifact (along polar directions).

Representative images from 4 of the 12 studied subjects are shown in Fig. 2, where the top panels show Cartesian images (arrows point to DRAs) and bottom ones are the corresponding radial images. All images correspond to a pre-defined early myocardial enhancement phase (see caption). Qualitative analysis (Fig. 2e) clearly shows the superiority of radial imaging in reducing the DRA. Similar findings were evident from quantitative assessment of the DRA maximal width (Fig. 2f).

## Conclusions

In this work, we demonstrated that radial imaging is capable of significantly reducing the dark rim artifact even in the early myocardial enhancement phase of a first-pass perfusion image series, due to its inherent robustness to Gibbs ringing. Such artifacts may confound interpretation and diagnosis of subendocardial perfusion defects (which may "fill in" early during the myocardial enhancement phase). Advanced (e.g., model-based/iterative) reconstruction techniques with radial acquisition can be used to improve image quality while preserving the described dark-rim-minimizing properties.

## Funding

Grant sponsors: American Heart Association Postdoctoral Fellowship Award 11POST7390063; National Institutes of Health grants nos. NHLBI HL38698, HL091989, N01-HV-68161, N01-HV-68162, N01-HV-68163, N01-HV-68164, U01 HL649141, and NIH CTSI UL1TR000124.
